# Point of Care Testing for SARS-COV-2 Antibodies before doing Endoscopy

**DOI:** 10.12669/pjms.39.2.5659

**Published:** 2023

**Authors:** Abeer Altaf, Zaigham Abbas, Muhammad Ali Qadeer, Mehreen Siyal

**Affiliations:** 1 Abeer Altaf, MBBS., Department of Hepatogastroenterology, Dr. Ziauddin Hospital, Clifton, Karachi, Pakistan; 2 Zaigham Abbas, MBBS, FCPS, FRCP, FACP, FACG, AGAF, FRCPI., Department of Hepatogastroenterology, Dr. Ziauddin Hospital, Clifton, Karachi, Pakistan; 3 Muhammad Ali Qadeer, MBBS., Department of Hepatogastroenterology, Dr. Ziauddin Hospital, Clifton, Karachi, Pakistan; 4 Mehreen Siyal, MBBS., Department of Hepatogastroenterology, Dr. Ziauddin Hospital, Clifton, Karachi, Pakistan

**Keywords:** COVID-19, Endoscopy, Seroprevalence, Cirrhosis, Asymptomatic

## Abstract

**Objectives:**

COVID-19 has taken the world by storm, creating much disparity among both healthcare and non-healthcare centres regarding the provision of services. The purpose of our study was to see the prevalence of the SARS-COV-2 exposure in the asymptomatic patients undergoing the endoscopic procedure, already triaged based on history and examination.

**Methods:**

Total 207 patients were enrolled during a time period of five months during October 2020 to April 2021 at Dr. Ziauddin Hospital Clifton campus, Karachi. In this prospective observational study patients undergoing endoscopic procedures were included after taking informed consent. The patients who already tested positive for COVID-19 by PCR were excluded. Patients were tested for Covid serology by immunochromatographic rapid serology *test* (ICT). Standard Operating Procedures for dealing with endoscopy patients during the COVID era were followed in all patients irrespective of antibody status.

**Result:**

Total number of patients included was 207; males were 121 (58.5%). The mean age was 48.5 ± 17.55 (range 13 to 92). Forty eight patients (23.2%) were positive for either antibody suggesting exposure to the COVID-19 virus. Out of these combined IgM and IgG positivity was seen in 24 (11.5%), IgM mono antibody positivity was seen in 7 (3.38%) and 17 (8.21%) of the study population tested positive for IgG only. 15 out of 46 (32.6%) patients with chronic liver disease in the cohort were seropositive for COVID antibodies.

**Conclusion:**

About one-fourth of the patients undergoing the endoscopic procedure were tested positive for COVID antibodies of which a significant percentage had chronic liver disease. It stresses the need of observing standard precautions to prevent the spread of infection during these procedures, especially in the vulnerable population.

## INTRODUCTION

Since December 2019 the world has changed. A new mutant virus termed the SARS-COV-2 that had emerged from a city in Wuhan China has spread itself miles beyond affecting over 200 million lives to date worldwide.^1^ The infectivity, transmissibility, and the virus itself has been a medical dilemma, where scientists are studying the virus in real-time and discovering something new every day.

International borders have been shut, various cities across the globe have been put in lockdowns to curb the spread of the virus, halting all kinds of daily activities and leaving behind both the citizens and authorities finding their way through “the new norm”. The intention being is to “flatten the curve”; to reduce the infections and hospitalizations, and to prevent the healthcare systems from being overwhelmed. Unfortunately, doctors and paramedics remain at the receiving end of COVID-19 as they are three times more vulnerable to contract the disease than the general population,^2^ and up to 20% have already been infected with the disease.^3^

Due to this, many hospitals around the world have taken a step back from performing routine activities, like surgeries, clinics, and procedures, and replaced them with only those cases that require emergent care to reduce exposure. This approach has been supported by many gastroenterological societies as well.^4^,^5^ According to data on SARS-COV-2 transmission, health care workers during endoscopic procedures are exposed 4.66 times more than non-exposed health care workers.^6^ This is amenable to the fact that upper GI procedures, including gastroscopy, ERCP, and EUS, may help in aerosolization^7^ of the virus particles and the risk is further increased when the patient dry retches, sneezes, coughs and requires intubation. Hence the reason why endoscopy is seen as a high-risk procedure for COVID-19 spreads and guidelines recommend keeping procedures according to the asymptomatic prevalence in the jurisdiction along with the availability of PPE.^8^

The purpose of our study was to see the prevalence of the SARS-COV-2 exposure by point of care serological testing in the patients requiring endoscopic procedure, already triaged based on history and examination.

## METHODS

The study was conducted at the endoscopy department of Dr. Ziauddin Hospital, Clifton campus within a duration of five months during October 2020 to April 2021. This prospective, cross-sectional study included patients requiring endoscopy procedure, who are asymptomatic for COVID-19 infection. Patients with a positive PCR for COVID-19 and those with symptoms specific for COVID-19 i.e., fever, cough, Shortness of breath etc. were excluded from the study.

All procedures were performed with precautionary measures taken and the procedure was done wearing complete PPE irrespective of the results. COVID PCR was not a pre-requisite for endoscopy, however if a patient has had it done beforehand and if it was positive then the patient was deemed inappropriate for the study. Two separate consent forms were provided to the patient, one being the standard pre-procedural consent form and another was for the special pre-procedural serology testing form. Point of care testing for COVID-19 IgM and IgG antibodies was performed by indirect chromatography method using Genuri Novel Coronavirus (2019-nCoV) IgG/IgM test kit (Genuri Biotech Inc. China). The individuals who had pre-endoscopy COVID IgM positive got the procedure done, albeit in a complete PPE, and they were advised to get themselves tested for COVID PCR. Those individuals who did not give consent for pre-endoscopy serology testing were not returned and their procedures were done as well even without it. The point of care testing was funded by the department and was not charged to the patient.

The study was conducted after getting institutional ethics committee approval [Reference code: 2530820ZAGE]. Continuous variables were expressed as mean and standard deviation. Categorical data were expressed as percentages. Crosstabulation was done to identify the association and ratio of seropositivity among the variables. The statistical analysis was done on the IBM SPSS version 20.0.

## RESULTS

A total of 207 patients were included in the study, age ranging from 13 to 92, mean of 48.55 ± 17.36 of which 138 (66.7%) were more than 40 years old. Of the 207 patients, 121 were males (58.5%) and 86 were females (41.5%).

Serology was conducted in point care fashion, results of which are presented in Table-I. Overall COVID-19 serology was tested amongst the study population and 48 out of 207 were positive (23.2%) and serology was negative in 159 (76.8%). Of these males were 29 (60.4%%) and female were 19 (29.5%). Twelve (17.39%) patients with either positive serology were less than 40 years old, and 36 (26.09%) patients were older than 40 years of age. 33 (22.92%) of these patients underwent upper GI endoscopy, 4 (50%) patients had sigmoidoscopy, 5 (16.13%) had a colonoscopy and 6 (25%) of them had ERCP performed.

**Table-I:** Serology results in the study population.

Variable	Total	Positive	

		N	%
Male	121	29	23.97%
Female	86	19	22.09%
Age < 40	69	12	17.39%
Age > 40	138	36	26.09%
Upper Endoscopy	144	33	22.92%
Sigmoidoscopy	8	4	50.00%
Colonoscopy	31	5	16.13%
ERCP	24	6	25.00%
Liver Cirrhosis	46	15	32.61%

Thirty-One individuals tested positive for COVID-19 IgM (15%) and IgG was found in 41 individuals (19.8%). Out of these 48 individuals, IgM and IgG were collectively positive in 24 individuals, 17 of which had sole IgG antibody and seven tested positive for IgM only.

Liver cirrhosis was present in 46 (22.2%) patients undergoing endoscopic procedure, and of which 29 (63.04%) were males and 17 (36.9%) were females, 18 (39.13%) were of less than 40 years old age and 28 (69.86%) of them were older than 40. Upper GI endoscopy was performed on 42 patients, one patient had sigmoidoscopy, two had a colonoscopy and one patient had ERCP. Fifteen of them (32.6%) had positive COVID serology. Nine (19.6%) of the cirrhotic population had positive COVID-19 IgM, IgG positivity was present in 15 (32.6%) of the cirrhotic population and dual antibody positivity was seen in 9 (19.6%) individuals with liver cirrhosis.

## DISCUSSION

COVID-19 has reshaped the way the world works nowadays, where “work from home” has become the new norm, but can such practices be done in medical profession? Globally it is estimated that GI cancers account for approximately 26% of the global cancer burden and 35% of all-cause mortality.^9^ Apart from that, cirrhotic individuals require variceal surveillance. Endoscopic procedures here play a vital role in early screening and treatment and that is why such procedures cannot be kept on hold for longer periods, especially considering that the virus has now slowed down due to the vaccines but the battle has not yet won.

COVID-19 is mainly diagnosed via a patient’s symptomatology that leads to the ignorance of the many “asymptomatic” or silent carriers of the disease. The CDC estimates that 35% of COVID-19 cases are asymptomatic and 40% of transmissions occur before symptom onset.^10^

Serological testing helps to understand the disease burden among those said individuals. Such a study was carried out by Zaidi S et al in Karachi, Pakistan, during May-July 2020 testing 1675 samples of which 36% came out seropositive.^11^ Another similar study was done to see seroprevalence of COVID-19 among adult male healthy blood donors which showed the difference of seropositivity during the peak pandemic vs post-peak pandemic times (21.4% vs 37.7%).^12^ In our study, 23.2% of such asymptomatic patients were seropositive for either COVID-19 antibodies with the majority being males (23.9%) and over the age of 40 years (26.09%).

Even though our study population had a majority of males and over the age of 40, gender disparity and its implications on the disease of COVID-19 has been studied and published worldwide. Ali S et al conducted a study checking for COVID-19 seroprevalence and found that in their study population 60% males had positive IgG and 61.1% had positive IgM antibody.^13^ Recently a meta-analysis conducted on 3,111,714 cases was published in Nature revealed that male patients had three times the odds of being admitted in ICU (OR = 2.84; 95% CI = 2.06, 3.92) and higher odds of mortality (OR = 1.39; 95% CI = 1.31, 1.47).^14^ Increasing age is another risk factor for COVID-19, younger individuals usually developing mild to no symptoms at all, and the spectrum of the disease increasing with the increase in age. A global meta-analysis concluded that case related mortality increased with age with the largest increase seen in patients aged 60-69 years compared to individuals less than 60 years of age (odds ratio 3.13, 95% confidence interval 2.61-3.76).^15^

In our study, 32.6% of them has liver cirrhosis who tested positive for either antibody, nine whom had positive IgM and 15 had the presence of IgG antibody. The chronic liver disease itself poses an altered immunity in the patients, hence such individuals are always vulnerable to catching infections and decompensating. The APCOLIS study concluded that the Child-Turcotte Pugh score of nine or higher was predictive of higher mortality [AUROC 0.94, HR = 19.2 (95 CI 2.3–163.3), p < 0.001, sensitivity 85.7% and specificity 94.4%)].^16^ The same study also stated that patients with cirrhosis had more instances of acute liver failure (32.6% vs 20%, p < 0.001) and development of new acute onset of liver injury during hospital stay (39.5% versus 7%, p < 0.001). Cirrhotic individuals also had a complicated hospital course with the development of COVID-19 related complications such as acute kidney injury (18.6% versus 5.4%, p < 0.001), respiratory failure (23.2% versus 8.6%, p < 0.001) and hypotension (14% versus 3.8%, p < 0.001). Another systemic review revealed that patients with CLD had significant COVID-19 clinical severity than non-CLD population (pooled OR 1.48 [95% CI 1.17, 1.87]; *p* = 0.001; I^2^ = 10%), more of such patients requiring machinal ventilation (pooled OR 2.22 [95% CI 0.67, 7.42]; *p* = 0.19; *I*^2^ = 36%) and pooled mortality was higher in patients with chronic liver disease as a co-morbid (pooled OR 1.78 [95% CI 1.09, 2.93]; *p* = 0.02; *I*^2^ = 0%).^17^

In our study, upper GI endoscopy was the most prevalent procedure being at 69.6%, followed by sigmoidoscopy (39%), colonoscopy (15%) and ERCP being at 11.6%. Of the 48 seropositive individuals, 39 had upper GI procedures whereas nine had lower GI procedures. Upper GI endoscopy procedures (endoscopy and ERCP) are regarded as aerosol-generating procedures, with the presence of viruses collected in samples of airway secretions.^18^ There is considerable debate as to whether the lower GI procedures (sigmoidoscopy and colonoscopy) even though being considered aerosol generating, pose the same risk of infection as the upper GI ones. There are studies that have demonstrated that RT-PCR has been positive in stool samples several days after testing negative in the nasal or throat swabs due to the high levels of ACE-2 receptors in the human intestines, the functional reservoir for the virus. It is a debatable question whether the virus is transmissible or not. A small study concluded that while the virus was found in the stool, the live or viable virus was not present.^19^ This finding was backed by another study which stated that the virus was inactivated by colonic fluids.^20^ Such studies conclude that lower GI procedures possess lower risk as compared to upper GI procedures hence not requiring a higher level of care while patient handling and using low-risk PPE.^21^

### There are few take home messages from this study:

- The usage of point of care COVID-19 serology for estimation of asymptomatic disease burden.
- The rapidity and cheaper option make it easier for screening patients who have no symptoms for COVID-19 before endoscopy especially in a resource-deprived country.
- Higher level of care while patient handling in individuals who are male, age greater than 40, who have liver cirrhosis or who have upper GI procedures to reduce cross infectivity and virus transmissivity in such high-risk patient population.

### Limitations

By testing patients only for serology, there was a higher chance of including individuals who tested falsely negative or were in their very early stage of infectivity where an RT-PCR could have detected them. Sample size being smaller, and single center, with the majority of individuals having undergone upper GI procedures, gave our study a confounding bias. Had there been a larger sample population with equal patients in both upper and lower GI procedure arms, and checking the samples of respiratory and stool for the presence of the virus, results would have shown a clearer picture of which procedure carried a higher risk of transmission and infection.

## CONCLUSION

With the commencement of the new decade, it is important to identify that even asymptomatic individuals are also active spreaders of the virus. As per our study, seroprevalence for COVID-19 was noted in such “high risk” population which included the males (23.9%), elderly (26.09%), those suffering from liver cirrhosis (32.61%) who are undergoing an endoscopy (22.92%) or ERCP (25%). Recognition of this section of patient population would help to prevent the spread of infection and observe necessary precautions during and after procedures and hopefully break the cycle of transmissibility.

### Authors’ Contribution:

**AA & ZA:** Did conceive, designing, statistical analysis and editing of manuscript.

**AA:** Did data collection and manuscript writing.

**MAQ & MS:** Did data collection, manuscript review and editing.

**ZA:** Did final approval of manuscript.

**AA & ZA:** Are responsible for accuracy and integrity of the work.
